# Adaptive Algorithm for Fast 3D Characterization of Magnetic Sensors

**DOI:** 10.3390/s25040995

**Published:** 2025-02-07

**Authors:** Moritz Boueke, Johannes Hoffmann, Mark Ellrichmann, Robert Bergholz, Gerhard Schmidt

**Affiliations:** 1 Department of Electrical and Information Engineering, Faculty of Engineering, Kiel University, 24143 Kiel, Germany; mobo@tf.uni-kiel.de (M.B.); jph@tf.uni-kiel.de (J.H.); 2 Department of Internal Medicine I, University Hospital Schleswig-Holstein, Campus Kiel, Kiel University, 24105 Kiel, Germany; mark.ellrichmann@uksh.de; 3 Department of Pediatric Surgery, University Hospital Schleswig-Holstein, Campus Kiel, Kiel University, 24105 Kiel, Germany; robert.bergholz@uksh.de

**Keywords:** magnetic sensor characterization, estimation algorithms, adaptive filters, NLMS control, magnetic field steering, impulse response, frequency response, directivity

## Abstract

Magnetic sensors are highly relevant in clinical and industrial applications such as localization tasks and geological investigations. The spatial behavior of these sensors is of great interest for accurate forward modeling and the consequential possibilities for sophisticated applications, e.g., solutions to inverse problems. In this contribution, we present a novel characterization approach using adaptive system identification approaches. We utilize a gradient-based algorithm for estimating impulse and corresponding frequency responses for a directivity analysis in 1D, 2D, and 3D. For this, we built a triaxial Helmholtz coil setup to generate a 3D directive field. This is controlled by an algorithm that exploits similarities in sensor behavior with respect to small differences in excitation field angles. We found advantages for a controlled adaptation, with faster convergence and a smaller system distance between estimations and measurements with a proposed control based on the contraction–expansion approach (CEA). With runtimes averaging less than 1.5 s per direction for full impulse response estimation, this proof of concept shows the potential of the proposed algorithm for enabling a feasible frequency and directivity characterization method.

## 1. Introduction

Magnetic measurements are becoming increasingly important in many areas, such as health care [1,2,3,4], indoor localization [5,6,7], or the investigation of geological conditions of the subsurface [8,9,10]. For many of these applications, a detailed forward model of the magnetic sensor is essential for gaining the best possible results when solving inverse problems. In these, a solution for, e.g., a source distribution should be determined by computations based on measured sensor observations. Thus, a characterization of the utilized magnetic sensors is necessary and can be achieved in various ways. The most common evaluation of the sensor includes the impulse or frequency response, a linearity curve, and the sensitivity [11]. The frequency response is the spectral transformation of the impulse response, both of which are used in this paper. Magnetic sensors usually measure one-directional projections of magnetic fields, which means they have a certain directional characteristic (vector magnetometers). This should be considered in a comprehensive characterization of the sensor as well [12].

There exist a variety of established magnetic sensor concepts. Scalar magnetometers like total field optically pumped magnetometers (OPMs) do not need a directional characterization by design; for other vector sensors, e.g., superconducting quantum interference devices (SQUIDs), it is not feasible to use these in a limited characterization setup due to cooling requirements. Otherwise, the directional response of vector magnetometers should be identifiable. Magnetoelectric (ME) sensors, for example, might profit from this, as the sensitive axis of the sensing element does not always coincide with the geometry [13,14]. Similar problems might occur with other sensor types based on the magnetoresistive (MR) effect or fluxgate magnetometry and should be identified and addressed, especially in areas like array applications where directionality is critical.

The directivity of a vector magnetometer can, for example, be assessed by measuring the normalized voltage response of the sensor to a defined magnetic field rotating around the sensing volume [12]. However, this does not account for the whole frequency response of the sensor in different directions. This is captured by the (spatially dependent) frequency response, which usually takes some time for the measurement of a single direction. It is nevertheless of interest to acquire the frequency response of a sensor in all directions to obtain an idea of the limitations and possibilities of a given sensor.

**Figure 1:**
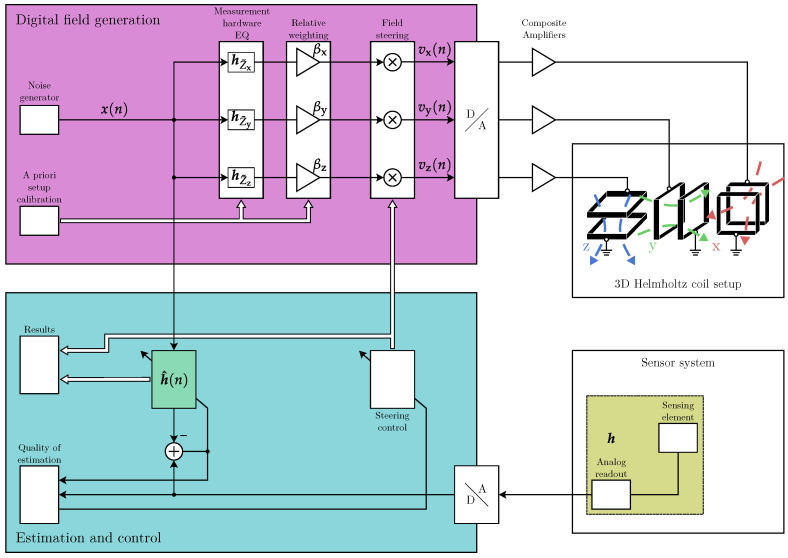
Block diagram of overall characterization system.

A fast characterization of sensors by means of a controlled adaptive algorithm has already been established for underwater transducers [15]. Some adjustments are necessary to apply this method to magnetic sensors for fully automated three dimensional (3D) characterization. In this contribution, we present a fast and robust algorithm for 3D characterization of a magnetic sensor system by estimating the direction-dependent impulse response. The sensor system incorporates the direct analog readout circuitry, as we are interested in characterizing the working behavior of the system and not the sensing element itself. Sensitive-axis frequency responses are typically measured with sine sweeps or noise measurements over a longer period, e.g., in a Helmholtz coil setup. A direct extension of such a measurement concept is often not feasible due to high time requirements and vulnerability to error when it comes to manual modification of the sensor systems alignment. Our proposed method tries to solve these issues. It is schematically visualized in Figure 1.

The remainder of this paper is structured as follows: In Section 2, the used hardware setup for characterization as well as the algorithm developed for fast characterization of sensors are presented. Section 3 displays the results of multiple measurements conducted with a fluxgate sensor. Discussion of these follows in Section 4. Lastly, a short conclusion is given in Section 5.

## 2. Materials and Methods

In the following, we describe the overall characterization system investigated in this contribution (cf. Figure 1). We distinguish the system components for magnetic field generation and those for the adaptive estimation of the sensor characteristics.

### 2.1. Field Generation

The field generation for the system is comprised of hardware components and of the software-controlled desired excitation signals.

**Figure 2:**
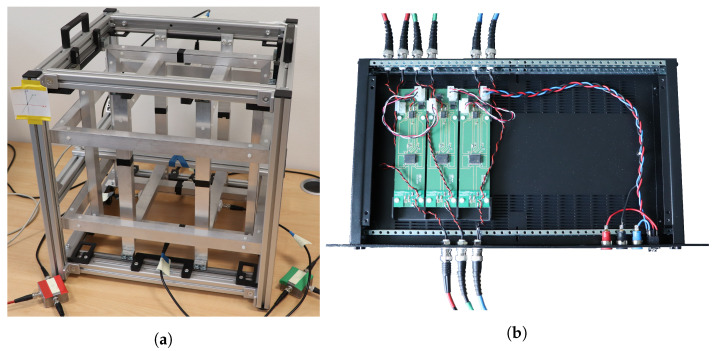
Custom hardware setup components used for measurements in this contribution. (**a**) Helmholtz coils used for field generation in characterization system. (**b**) Rack-mountable composite amplifiers for coil excitation.

With the underlying idea of steering a known magnetic field towards the sensor under characterization from arbitrary directions, a triaxial Helmholtz coil setup was chosen and built for the excitation. A picture of this is shown in Figure 2a.

There are three orthogonal pairs of aluminum enclosures that are nested within one another. These pairs each form individual Helmholtz coils, which are regularly used to produce approximately homogeneous magnetic fields in their center area [16]. Here, we chose to work with the common change in form factor from cylindrical coils to square ones, which is well established [17].

The individual coils have $N_{\mathrm{coil}}=60$ windings of enameled copper wire, each with a conductor diameter of 0.8 mm, which corresponds to a 0.5 mm^2^ cross-section. After assembly, we defined the inner coil as producing a magnetic field along the x-axis, the mid coil that as associated with the y-axis, and the outer coil that as associated with the z-axis. We also measured some parameters of the resulting practical setup. Regarding static quantities, we measured the resistances and the edge lengths *ℓ* of the individual coils, the results of which are summarized in Table 1. For each individual coil of a pair of coils, the values are identical.

We used a combined digital–analog and analog–digital converter (DAC and ADC, respectively; model Fireface UFX+; RME [18]) operated at sampling rate $f_{s}=48$ kHz in combination with a low-noise composite amplifier (based on [19]) to excite the coils over the three channels—one for each direction (x, y, and z). Each output channel was split afterwards and connected to both coils of the respective direction with coaxial cables. The supply voltage for the rack-mountable amplifier unit shown in Figure 2b is provided by a laboratory power supply (GPP-4323; Good Will Instrument Co., Ltd., Taipei, Taiwan [20]).

In addition to the (real-valued) resistances of the coils, we had to consider the frequency-dependent impedances to properly generate a desired magnetic field. We derived the equalizers (EQs) for impedance consideration from noise excitations of each coil pair. Therefore, we generated and recorded uniformly distributed white noise, which is used as the DAC input and propagated to the amplifiers. With the current sensing modules of the amplifiers, we recorded the current flow over a 100 mΩ shunt resistor. In each coil pair measurement, we computed the power spectral density (PSD) $S_{vv}\left(\Omega \right)$ of the voltage signal as well as the cross power spectral density (cross PSD, CPSD) $S_{vi}\left(\Omega \right)$ of the current and voltage signals. Time signals of 90 s were recorded, with 1 s discarded at the start and end to avoid the impact of possible transient behavior. The current signals were scaled by $\frac{1}{100\;m\Omega }=10\frac{1}{\Omega }$ to account for the shunt resistance.

As parameters for the PSD calculation, the size of the fast Fourier transform (FFT) was set to about a second with an even number of frequency bins for the filter design. We used a Hann window of the same length as the FFT and a 50% overlap between the signal sections. From these calculations, the impedance Z(Ω) for a given coil measurement can be derived as the cross power spectral density $S_{vi}\left(\Omega \right)$ normalized by the power spectral density $S_{vv}\left(\Omega \right)$. Since we only used the impedances’ magnitude information for our equalizer design, we calculated this from the complex valued results as follows:

(1) $$\left|Z(\Omega )\right|=\left|\frac{S_{vi}\left(\Omega \right)}{S_{vv}\left(\Omega \right)}\right|.$$

For their use as filter design target curves, we smoothed the results along the frequency axis with a basic infinite impulse response (IIR) filter (Butterworth design, filter order 4). The very low frequencies were not very well captured by our ADC; thus, we manually set the values for the first 5 Hz to the coil pair resistances of 5 Ω, 6 Ω, and 7 Ω, respectively. Furthermore, we limited the EQ design to a frequency range of interest according to the sensor’s specifications. As we used a fluxgate sensor (FLC 100, Stefan Mayer Instruments, Dinslaken, Germany [21], powered by the GPP-4323) with a sensitive frequency range of 0 kHz to 1 kHz to test our presented method, we limited the design curves for the coil equalizing filters to 2 kHz, with the signal components above remaining unchanged. Since the impedance grows with higher frequencies and, correspondingly, lower frequencies become dampened in the equalization approach, this choice of a bandwidth allows for higher-signal components in the frequency area of interest.

**Figure 3:**
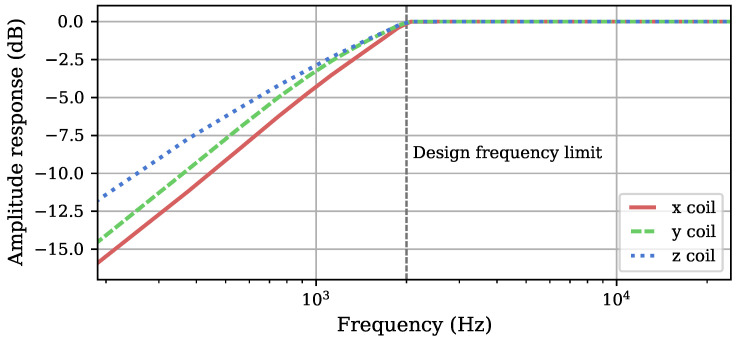
Magnitude of the different unity gain equalization filters. The frequency axis is scaled logarithmically as the chosen design limit up to which the impedances are emulated (the marked 2 kHz), while measurement was performed up to $\frac{f_{s}}{2}=24$ kHz.

The individual target curves are normalized to their respective maxima $Z_{x,0}$, $Z_{y,0}$, and $Z_{z,0}$ to obtain filters with unity gain at the maximum amplitude. With the resulting specified target characteristics, we designed type I linear phase finite impulse response (FIR) filters $h_{{\tilde{Z}}_{x}}$, $h_{{\tilde{Z}}_{y}}$, and $h_{{\tilde{Z}}_{z}}$ of order $N_{\mathrm{filter}}=256$ by means of a least squares approach [22,23]. The resulting magnitude behavior of the filters is visualized in Figure 3.

We needed to include an additional gain correction step after imprinting the impedance behavior with the equalizing filters to harmonize the magnetic flux densities to the coil sizes. For the derivation of these gains, we consider the general equation for the magnetic field at the center of a square Helmholtz coil with the vacuum magnetic permeability $\mu_{0}$, the (time-variant) current through the coil i(t), the equivalent cylindrical Helmholtz coil’s radius $R_{H}$, and the voltage (noise) amplitude $u_{0}$, which is the same for the different coils. When a specific coil’s quantity is described, the respective Cartesian direction is used in the variable’s indices. Thus, we obtain the equation

(2) $$\begin{array}{cc} B_{x}\left(0,t\right) & =\frac{8}{\sqrt{125}}\frac{\mu_{0}N_{\mathrm{coil}}i_{x}\left(t\right)}{R_{H}} \end{array}$$

(3) $$\begin{array}{cc} \; & \propto \frac{\mu_{0}N_{\mathrm{coil}}i_{x}\left(t\right)\sqrt{\pi }}{\ell_{x}} \end{array}$$

(4) $$\begin{array}{cc} \; & \propto \frac{u_{0}}{Z_{x,0}\ell_{x}} \end{array}$$

to describe this field for the x coil at the center of the pair. We use this coil as the reference and thus set its additional gain to $\beta_{x}=1$. The gains for the y and z coils follow from equating the different parameter versions for (4) as

(5) $$\begin{array}{cc} \; & \beta_{x}\frac{1}{Z_{x,0}\ell_{x}}=\frac{1}{Z_{x,0}\ell_{x}}\overset{!}{=}\beta_{y}\frac{1}{Z_{y,0}\ell_{y}}\overset{!}{=}\beta_{z}\frac{1}{Z_{z,0}\ell_{z}} \end{array}$$

(6) $$\begin{array}{cc} \; & \Rightarrow \beta_{y}=\frac{Z_{y,0}\ell_{y}}{Z_{x,0}\ell_{x}},\;\beta_{z}=\frac{Z_{z,0}\ell_{z}}{Z_{x,0}\ell_{x}}, \end{array}$$

which are quantified for our setup with the chosen replicated frequency area in Table 2.

The described hardware together with the equalization procedure then enables the generation of very similar individual homogeneous fields with the different coils using the same excitation signal. This, in turn, means that we are able to produce arbitrarily directed fields by defining an elevation and azimuth angle—θ and φ, respectively. These are relative to the center of the coil system and transformed from these spherical coordinates to Cartesian coordinates to scale the excitation signal for the individual coil channels. For this, we chose a uniformly distributed white noise signal x(n) to enable characterization of all sensing frequencies at once. With specific given angles $\theta_{0},\;\varphi_{0}$, this leads to the coil channel excitation signals

(7) $$\begin{array}{cc} v_{x}\left(n\right) & =\beta_{x}\left(h_{{\tilde{Z}}_{x}}*\left[x\left(n\right)\sin\left(\theta_{0}\right)\cos\left(\varphi_{0}\right)\right]\right), \end{array}$$

(8) $$\begin{array}{cc} v_{y}\left(n\right) & =\beta_{y}\left(h_{{\tilde{Z}}_{y}}*\left[x\left(n\right)\sin\left(\theta_{0}\right)\sin\left(\varphi_{0}\right)\right]\right), \end{array}$$

(9) $$\begin{array}{cc} v_{z}\left(n\right) & =\beta_{z}\left(h_{{\tilde{Z}}_{z}}*\left[x\left(n\right)\cos\left(\theta_{0}\right)\right]\right), \end{array}$$

which are calculated programmatically and then passed as voltages to the DAC. Practically, we designed the software system to calculate the desired directions and resulting Cartesian weights automatically from given inputs. When a single direction (1D) is desired, this is typically measured in-plane with the plate in the center of the coils and a singular offset angle can be introduced. This is also possible for a planar 360° (2D) characterization around a fixed axis (e.g., z-axis) with a number of equal, discrete angular steps $N_{\varphi }$. The reference axis for both 1D and 2D characterization can also be changed. In a full 3D characterization scenario, the number of discrete steps can be set for azimuth and elevation ($N_{\theta }$) separately. The used angle pairs are derived from the division of 360° and 180°, respectively, which results in a total number of directional steps of $N_{\mathrm{steps}}=N_{\theta }N_{\varphi }$.

### 2.2. Adaptive Filter

**Figure 4:**
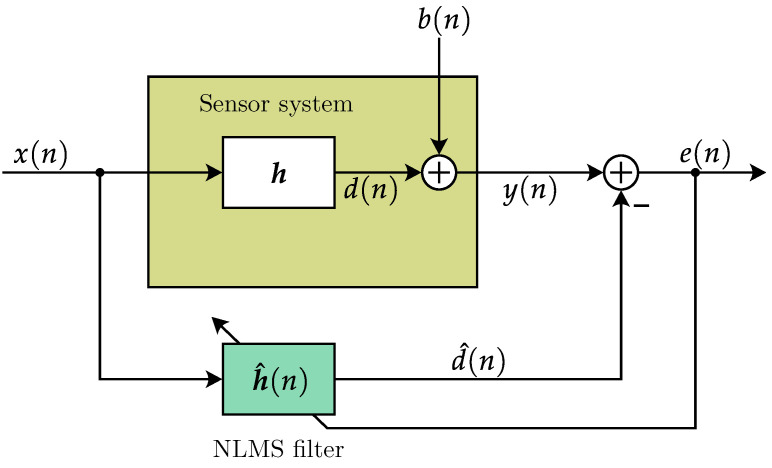
Adaptive filter used for system identification.

In this contribution, we assume a high similarity between sensor system responses to magnetic excitation from similar directions. We want to exploit this by initializing a directional response estimate with the last finished estimate from an orientationally close excitation. For that, we use a normalized least mean squares (NLMS) algorithm to estimate the sensor’s impulse response

(10) $$h={\left[\begin{array}{c} h_{0},h_{1},\ldots ,h_{N-1} \end{array}\right]}^{T}$$

of length *N*. We assume a maximal filter length that can capture the full sensor behavior. Our goal of a precise impulse response estimation is a system identification task for which a generalized representation based on [24] is shown in Figure 4. Here, idealized digital–analog conversions and vice versa are assumed. This is also assumed in the following derivations to highlight the parallels between the physical system and the estimated model taken advantage of by the NLMS.

The sensor produces the ideal sensor output d(n) due to excitation with the signal x(n), which is connected via the impulse response by the relation

(11) $$d\left(n\right)=h^{T}x\left(n\right).$$

The respective signal vector x(n) consists of the last *N* samples of the corresponding time signal. This ideal output is then superimposed by some noise b(n) from various sources (e.g., magnetic (Barkhausen) noise, electromagnetic noise from circuit components such as amplifiers and superimposed interference signals, 1/f noise, mechanical noise [25,26]) to produce the measured output signal

(12) y(n)=d(n)+b(n).

The adaptive filter $\hat{h}\left(n\right)$ is excited with the same signal x(n) and provides an estimated output signal

(13) $$\hat{d}\left(n\right)={\hat{h}}^{T}\left(n\right)x\left(n\right),$$

which ideally should reproduce the measured output for the sensor. Correspondingly, the error for this adaptive filter is defined as

(14) $$e(n)=y\left(n\right)-\hat{d}\left(n\right),$$

with a small error indicating a good adaptation of the filter to the actual sensor system (including noise); thus, the error is a good estimate of the sensor characteristics for the chosen excitation. Only real-valued time signals are considered in this work, so notation considering generally possible complex-valued signals is omitted. We also use a reference channel to eliminate the influence of digital–analog conversions and vice versa in the overall system (cf. Figure 1) by using a direct feedback channel in addition to the coil excitation channels on the output and the sensor voltage at the input.

We use the adaptive identification approach with the minimization of the expected value of the system distance between the real sensor and the estimated system

(15) $$E\left\{{∥\Delta h(n)∥}^{2}\right\}=E\left\{{∥h-\hat{h}\left(n\right)∥}^{2}\right\}$$

as our optimization target over time [24]. The adaptation procedure is generally based on a gradient-based incremental linear change to the current filter estimate $\hat{h}\left(n\right)$ from one time step to the next. Thus, a general update of the filter coefficients can be described with the time-varying scaling step size term μ(n) and a correction term $\Delta_{\mathrm{corr}}\left(x(n),(n)\right)$ depending on the excitation signal vector and error signal as

(16) $$\hat{h}\left(n+1\right)=\hat{h}\left(n\right)+\mu \left(n\right)\;\Delta_{\mathrm{corr}}\left(x(n),(n)\right)$$

with the correction depending on the chosen adaptation scheme. Our choice for this scheme, as mentioned above, is the NLMS algorithm, which extends this correction term to

(17) $$\Delta_{\mathrm{corr}}\left(x(n),(n)\right)=x\left(n\right)\frac{\left(n\right)}{{∥x(n)∥}^{2}}.$$

We cannot influence the update term directly, as it only depends on the (random) excitation signal vector, which we define as the last *N* samples of the discrete signal used for the field generation calculations (see Section 2.1) and the resulting error signal. The normalization employed is relative to the excitation signal vector’s norm, which can be efficiently calculated by the recursion

(18) $${∥x(n)∥}^{2}={∥x(n-1)∥}^{2}-x^{2}\left(n-N\right)+x^{2}\left(n\right),$$

as described in [24] with a robust implementation that we used as a template [27].

To tune the adaptive filter towards the desired behavior (a minimal remaining system distance with fast convergence), we can instead influence the filter update in (16) via the step size parameter. An alternative would be the control of the filter behavior relying only on a regularization term as the divisor to the correction term $\Delta_{\mathrm{corr}}\left(x(n),(n)\right)$ or a combination of both approaches [24]. Since these are mutually convertible and previous work uses step size control [15], we also choose a step-size-only approach.

For a stable update behavior of the algorithm, the step size has to be between 0 and 2. In the most naïve approach, we can use a constant step size $\mu \left(n\right)=\mu_{\mathrm{fix}}$. A step size close to, but not exceeding, 1 leads to a fast adaptation with the trade-off of a comparatively higher system distance. Conversely, a small step size (close to 0) can achieve a smaller final system mismatch over a longer adaptation time [24].

Time varying step size control approaches try to mitigate this trade-off by using a large step size at first for a fast convergence and by gradually reducing the factor to arrive at a more desirable system distance. In contrast to [15], we do not consider a remaining system component as a part of the sensor system noise, since the decay behavior is assumed to be much faster in the electromagnetic domain in contrast to (underwater) acoustics.

We get a pseudo-optimal variable step size based on the mean power of the undisturbed error $e_{u}\left(n\right)$ in the form of

(19) $$\mu_{\mathrm{opt}}\left(n\right)\approx \frac{E\left\{e_{u}^{2}\left(n\right)\right\}}{E\left\{e^{2}\left(n\right)\right\}}$$

as derived in [28]. The undisturbed error describes the actual difference between the ideal sensor output and the filter output signal

(20) $$e_{u}\left(n\right)=d\left(n\right)-\hat{d}\left(n\right).$$

When taking into account practical implementations, we can neither use expectation operators as in (19), nor have knowledge of the ideal sensor output. Thus, we use short-term smoothing for the estimation of the expected values. Our smoothing, denoted by an overline, uses a two-parameter IIR filter approach [24]: the power of an example signal s(n) is combined with the smoothing parameter γ(n) according to

(21) $$\bar{s^{2}\left(n\right)}=\left(1-\gamma (n)\right)\;s^{2}\left(n\right)+\gamma \left(n\right)\;\bar{s^{2}\left(n-1\right)}.$$

To account for differences in smoothing behavior for rising and falling signal power, the smoothing parameter is set to a rising constant $\gamma_{r}$ if the instantaneous power exceeds the previously smoothed short-term power and to a different falling constant $\gamma_{f}$ otherwise:

(22) $$\gamma \left(n\right)=\left\{\begin{array}{cc} \gamma_{r}, & \mathrm{if}\;s^{2}\left(n\right)>\bar{s^{2}\left(n-1\right)},\\ \gamma_{f}, & \mathrm{else}, \end{array}\right.\;0<\gamma_{r}<\gamma_{f}<1.$$

In addition to this, we split up the undisturbed error power into excitation power and the estimated system distance with a new symbol Γ(n) [15]. This leads to the general variable step size

(23) $$\hat{\mu }\left(n\right)=\frac{\;\bar{e_{u}^{2}\left(n\right)}\;}{\bar{e^{2}\left(n\right)}}=\frac{\bar{x^{2}\left(n\right)}\Gamma \left(n\right)}{\bar{e^{2}\left(n\right)}},$$

which is now only missing the system distance estimate. We employed two different methods for this estimation, which will now be described.

The first method is dead-time (DT) coefficient-based system distance estimation. Here, we assume that the length of the target impulse response is shorter than the (accordingly) chosen length of the adaptive filter *N*. Consequently, a number of coefficients $N_{\mathrm{DT}}$ at the end of the adaptive filter $\hat{h}\left(n\right)$ should be zero for a correct system identification as

(24) $$\left[\begin{array}{c} h_{N-N_{\mathrm{DT}}-1},h_{N-N_{\mathrm{DT}}},\ldots ,h_{N-1} \end{array}\right]=0$$

is assumed. Since the estimation error for an adaptive filter is spread equally among its coefficients [24], an estimate of the system distance can be calculated according to

(25) $${\hat{\Gamma }}_{\mathrm{DT}}\left(n\right)=\frac{N}{N_{\mathrm{DT}}}\sum_{j=N-N_{\mathrm{DT}}-1}^{N-1}{\hat{h}}_{j}^{2}\left(n\right),$$

as introduced for artificial delays in [28].

In addition to employing this approach as a standalone solution for the estimation of the system distance, the result is utilized as the initial value for the contraction–expansion approach (CEA) for step size control (cf. [15]). For this, we use the contraction parameter $A\left(\mu ,N\right)$ describing the theoretical adaptation in undistorted conditions, defined for our case as

(26) $$A\left(\hat{\mu }\left(n\right),N\right)=\frac{{\hat{\mu }}^{2}\left(n\right)}{N}-\frac{2\;\hat{\mu }\left(n\right)}{N}+1,$$

and the expansion parameter $B\left(\mu ,N\right)$, both formulated only with regard to step size (without regularization), as

(27) $$B\left(\hat{\mu }\left(n\right),N\right)=\frac{{\hat{\mu }}^{2}\left(n\right)}{N},$$

which encompasses the influence of noise on the adaptation process [24]. Together, they enable the description of a propagating system distance estimate ${\hat{\Gamma }}_{\mathrm{CEA}}\left(n\right)$ depending on the previous estimation, the power of the excitation signal estimated by its smoothed short-term value, and the estimated noise power ${\hat{\sigma }}_{b}^{2}$, formulated as

(28) $${\hat{\Gamma }}_{\mathrm{CEA}}\left(n\right)=A\left(\hat{\mu }\left(n-1\right),N\right)\;{\hat{\Gamma }}_{\mathrm{CEA}}\left(n-1\right)+B\left(\hat{\mu }\left(n-1\right),N\right)\frac{{\hat{\sigma }}_{b}^{2}}{\;\bar{x^{2}\left(n\right)}\;}$$

according to [15,24]. As mentioned above, the estimate ${\hat{\Gamma }}_{\mathrm{DT}}$ is used to initialize this estimation, since the approach only acts iteratively on this initial guess and should therefore be determined by a reasonable assumption given by the dead-time concept. The estimation of the noise power is achieved in a short, adjustable period of $N_{b}$ samples before the start of the actual adaptation process. For this, the excitation signal is set to zero and the smoothed sensor signal power is averaged over the given period:

(29) $${\hat{\sigma }}_{b}^{2}=\frac{1}{N_{b}}\sum_{k=0}^{N_{b}-1}\bar{y^{2}\left(k\right)}.$$

To avoid possible transient effects distorting the characterization at the start of excitation after this noise estimation period, there is also a short settling period of $N_{\mathrm{set}}$ samples afterwards in which the first excitation signal is active but before the adaptation process starts.

With all of these step size options, we have several thresholds and conditions to determine when which approach is used. Firstly, at the start time $n_{0}$ of adaptation from a new direction, we use a time-based threshold $N_{\mathrm{fix}}$ up to which we use a fixed step size $\mu_{\mathrm{fix}}$. This is to account for the known change of the overall system—that is, the change in the direction of the excitation field—which is bound to change the system behavior and thus lead to a sudden increase in the system distance. The fixed step size enables us to adapt to the new system behavior quickly and to enable the system distance estimate to readjust to new circumstances.

After that, we use the ratio $\bar{e^{2}\left(n\right)}\;/\;\bar{y^{2}\left(n\right)}$ between the smoothed error signal power and the smoothed sensor signal power as an indicator of convergence. When this falls below a threshold $\eta_{\mathrm{fix}}$, the system distance estimate ${\hat{\Gamma }}_{\mathrm{DT}}$ is used to determine the step size as follows:

(30) $${\hat{\mu }}_{\mathrm{DT}}\left(n\right)=\min\left\{1,\frac{\bar{x^{2}\left(n\right)}\;{\hat{\Gamma }}_{\mathrm{DT}}\left(n\right)}{\bar{e^{2}\left(n\right)}}\right\}.$$

Once we fall below a threshold $\eta_{\mathrm{DT}}$, we then initialize the CEA system distance ${\hat{\Gamma }}_{\mathrm{CEA}}$ and use it for the calculation of our step size:

(31) $${\hat{\mu }}_{\mathrm{CEA}}\left(n\right)=\min\left\{1,\frac{\bar{x^{2}\left(n\right)}\;{\hat{\Gamma }}_{\mathrm{CEA}}\left(n\right)}{\bar{e^{2}\left(n\right)}}\right\}.$$

**Figure 5:**
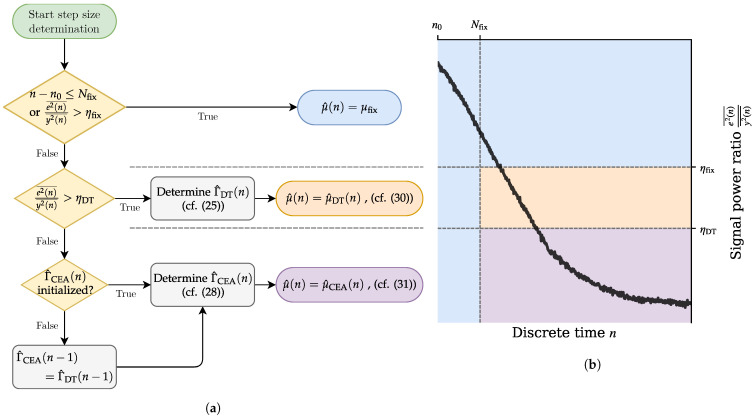
Visualization of the step size determination. The three possible step size outcomes are color-coded as blue for fixed step size, orange for dead-time (DT)-based step size, and purple for a step size based on the contraction–expansion approach (CEA) in both figure parts. (**a**) Flow chart of the threshold steps to determine the correct step size. (**b**) Example graph of a possible progression of the error/sensor ratio. The plot is purely illustrative and not based on real data.

In any case, we use a minimum operator around the time-variant step sizes to ensure a valid step size $\hat{\mu }\left(n\right)\leq 1$ even in situations where the estimates might produce a larger result. This overall control scheme is visualized in Figure 5.

On top of the different approaches to determine the step size, we have the added control dimension of the field steering (in contrast, e.g., to [15]). We aim to characterize the sensor impulse response in different directions, which is why we use the weighted excitations to generate a field in the current direction of interest. Thus, as mentioned above, we start with a noise evaluation period, followed by a settling period under excitation, and then the actual adaptation process begins for the first field orientation. The change of position is initiated only after full frames due to the way the excitation signals are driven via the DAC. The frameshift $N_{\mathrm{frame}}$ should therefore be chosen to be sufficiently small for a quick response to an orientation change event. Any orientation-control-related time thresholds as well as the periods for the noise power estimation and settling are rounded to the next full frameshift in the implementation accordingly. The different steering control thresholds are introduced in the following.

To avoid erroneous jumps to a next position due to behavior right after a directional change as well as the filter becoming stuck in a position for too long due to unsuitable parametrization, there are thresholds for a minimal and maximal time for each position, $N_{\min}$ and $N_{\max}$. For a quality assessment, we use the estimate ${\hat{\Gamma }}_{\mathrm{DT}}$, as this is grounded in the actual current filter coefficients, and use this to determine a sufficiently good convergence of the NLMS algorithm. When the threshold $\zeta_{\mathrm{suff}}$ is reached, we thus store the current filter estimate as our characterized impulse response for the present field direction and move to the next one.

A fallback is triggered if the average sensor power over a frame is too close—quantified by the threshold value $\zeta_{\mathrm{SNR}}$—to the estimated noise level, in which case the step to the next orientation is triggered as well. This is due to the fact that a directional sensor also has insensitive directions in which no sufficient system distance can be reached as the sensor signal does not carry enough information to arrive at a sensible estimate—the correct sensor response estimate would basically be zero (plus measurement noise). This can be formalized for a frame index *k* as the average frame estimate of the signal-to-noise ratio (SNR)

(32) $$\mathrm{SNR}\left(k\right)=\frac{\frac{1}{N_{\mathrm{frame}}}\sum_{i=0}^{N_{\mathrm{frame}}-1}\bar{y^{2}\left(k\;\cdot \;N_{\mathrm{frame}}+i\right)}}{{\hat{\sigma }}_{b}^{2}}.$$

To summarize, the switch to the next field direction is triggered when

(33) $$n-n_{0}>N_{\min}$$

holds and any of the conditions

(34) $${\hat{\Gamma }}_{\mathrm{DT}}\leq \zeta_{\mathrm{suff}},$$

(35) $$\mathrm{SNR}\leq \zeta_{\mathrm{SNR}},$$

(36) $$n-n_{0}>N_{\max}$$

is true.

At a new field orientation, the filter coefficients stay the same and function as an initialization for the new directional response. Since the change in direction is relatively small for a sufficiently detailed choice of orientation points, the change in the sensor response is assumed to be small and, as such, the initialization with the previous coefficients is reasonable.

## 3. Results

The following section first describes the general parameters and implementation details used to test the presented algorithm. We present different findings from analysis of estimation runs in one axis, two-dimensional planes, and fully 3D directional impulse response estimation. This structure is used to highlight and analyze different aspects of the algorithm in a scaling fashion.

### 3.1. Parameters and Realization Details

As mentioned in Section 2.1, we used an FLC 100 fluxgate sensor [21] for the proof of concept experiments performed in this work. To circumvent a significant influence of signal breakthrough at its self-excitation frequency around 13.5 kHz, we employed a digital notch filter around this frequency after analog–digital conversion with the used ADC. The filter is designed with its center frequency at 13.5 kHz, a bandwidth of 200 Hz, and a gain of −40 dB.

While some parameters were changed depending on the type of characterization to enable the analysis of specific workings of the algorithm, most were kept the same to provide comparability and transferability to the different experiments.

The parameters were chosen empirically based on experience gained during system development. Smoothing factors and thresholds are based on a trade-off between control response and estimation accuracy, and the filter length is set long enough to capture the full apparent impulse response.

The algorithm’s parameters are collected in Table 3.

### 3.2. 1D Estimation, Control Schemes and Step Size Behavior

First, we examined the algorithm without any influence of the orientation control—simply exciting one field direction in line with the assumed sensitive axis of the sensor under characterization. For that, we set the number of azimuth and elevation steps, $N_{\varphi }$ and $N_{\theta }$, respectively, to 1 and the time thresholds to $N_{\min}=N_{\max}=30$ s as shown in Table 3. As we wanted to investigate different control schemes, we performed four different runs of the experiment: two runs with fixed step sizes of $\hat{\mu }=1$ and $\hat{\mu }=0.1$, respectively, one run with a DT controlled step size, and one run with a CEA controlled step size. Our goal was a comparison of the resulting impulse response estimates and measuring the adaptation behavior with regards to the system distance and the step size.

**Figure 6:**
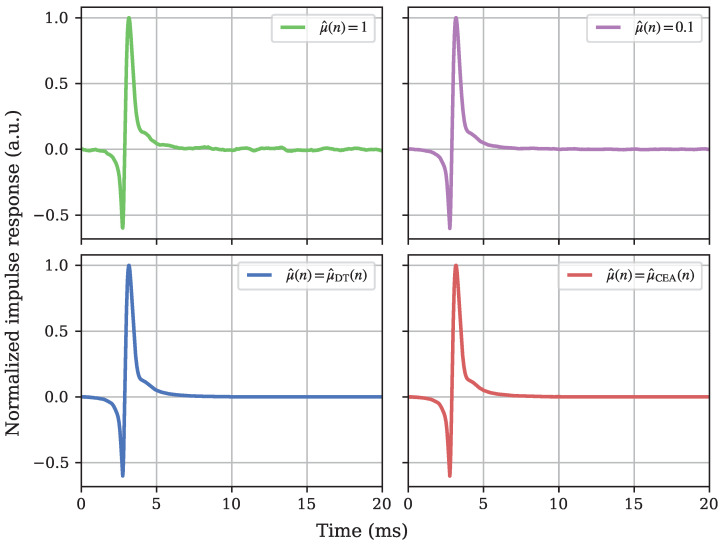
Comparison of the normalized impulse response estimation results after complete 1D adaptation runs. The more polished and smooth nature of the estimates resulting from adaptive step size control schemes is clearly observable, while a clear distinction is not visible between the DT and CEA controlled results.

The resulting normalized impulse response estimations are visualized in Figure 6. The upper left depicts the impulse response estimated with a large fixed step size, which clearly leads to a noisy estimate. The smaller fixed step size (upper right) reduces this erratic nature in the long tail somewhat; it is, however, still less smooth than the estimation results from the adaptively controlled runs. Between these, the DT (lower left) and CEA (lower right) control do not show a visually distinguishable behavior and show only minuscule differences in the exact timing of the impulse peak.

**Figure 7:**
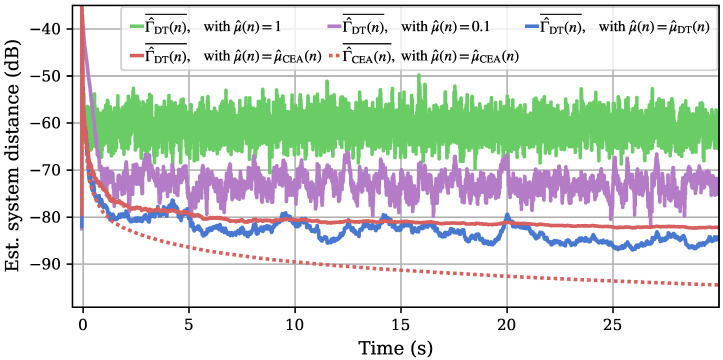
Adaptation plot of the estimated system differences from 1D measurements with different step sizes. As a reference, the DT estimates of the system distance with fixed step sizes of 1 (green) and 0.1 (purple) are shown. The former adapts fast but stays at a relatively imprecise system distance while the latter needs more time during the initial adaptation with a better ending plateau. DT (blue) and CEA (red) controlled adaptations lead to less of a plateau and generally smaller DT-based system distance estimates with a fast initial convergence. The CEA controlled measurement also shows the CEA-based system distance estimate as a dotted line.

Next, we look at the smoothed system distance estimates calculated by our algorithm (cf. Figure 7). The DT-based system distance estimation $\bar{{\hat{\Gamma }}_{\mathrm{DT}}\left(n\right)}$ is performed for all four experiments, as it is only based on the current impulse response approximation (cf. (25)). The CEA system distance $\bar{{\hat{\Gamma }}_{\mathrm{CEA}}\left(n\right)}$, on the other hand, is only derived when this control scheme is used. The experiment with a fixed step size of 1 (green) shows a steep convergence at the start of adaptation but stabilizes around a relatively large system distance of −60 dB. A smaller, fixed step size of 0.1 (purple) can achieve a lower remaining system distance after adaptation but it takes significantly longer for the first adaptation, observable from the much less steep convergence in the first second. Both adaptive control approaches achieve a similar convergence speed with the (fastest possible) fixed step size of 1; they are also able to surpass both fixed step size runs with their ongoing system distance estimations. The DT-controlled adaptation (blue) shows a higher fluctuation in its system distance estimate in comparison with the CEA control (red). The latter shows a deviation between the DT and CEA estimation for the system distance with the CEA estimate (dotted) achieving smaller modeled distances based on the initial behavior. Overall, both adaptive approaches led to significant improvement, with the model based on CEA showing potential in achieving lower remaining errors.

**Figure 8:**
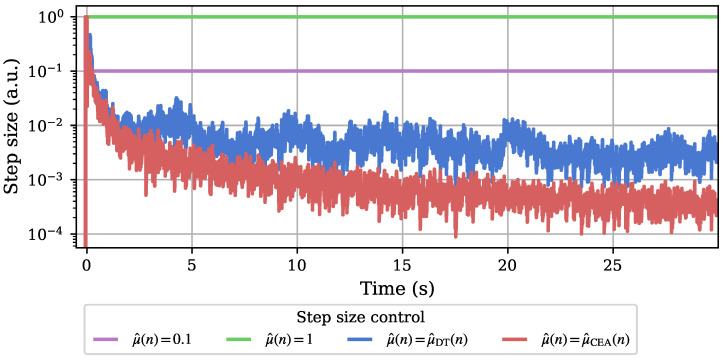
A comparison of the development of the different control step sizes over the course of the 1D adaptation runs. The fixed step sizes are plotted for comparison. A more erratic behavior of the DT-controlled step size at overall larger values at later stages of the adaptation compared to the CEA-based step size can be observed. The y-axis is scaled logarithmically for a more visible separation of the two adaptive step sizes’ behaviors.

The difference in the resulting step size behavior is visualized in Figure 8 with logarithmic scaling for better differentiation in small step size ranges. Both experiments with fixed step size obviously lead to constant behavior over the whole 30 s adaptation time frame. The adaptively controlled step sizes qualitatively resemble the different system distance estimates, which is expected due to their proportional calculation (cf. (30) and (31)). With the smaller step size at later adaptation times and an ever-decreasing modeled system distance estimate for CEA control, however, a smaller remaining error between system model and estimated physical sensor system is theoretically achievable [24]. This is of interest for the use case presented in this work.

### 3.3. 2D Estimation, Threshold Analysis, and Adaptation Behavior

With the 1D experiments investigated above, we heuristically tested and chose parameters for our proposed algorithm. In addition, a general proof of principle in the domain of interest was reached for the CEA control. Thus, we wanted to check the algorithm’s behavior for the 2D case, as it allows for simplified visualization compared to a 3D experiment. We aimed to gather information on the adaptation behavior of different positions investigated in the stepwise algorithm procedure as well as the respective step size behavior and the control of the direction index based on the proposed threshold approaches. Furthermore, we analyzed the resulting sensor response estimates from our experiment.

**Figure 9:**
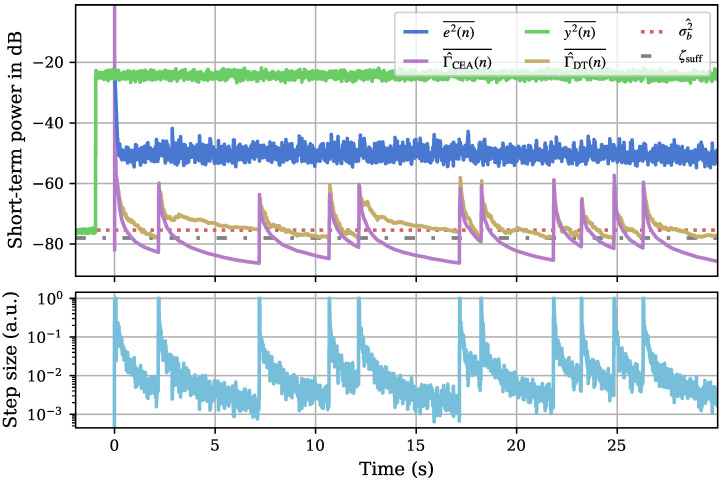
Visualization of the adaptation behavior over the starting period of a 2D adaptation measurement. The continuing, beneficial model adaptation is visible, as is the change in the system triggered by a sufficiently good estimate or a time-based threshold. A new direction’s short starting time with a step size of 1 can also be discerned from the progression of the adaptive step size in the lower plot controlled by the CEA.

To gain knowledge about the temporal behavior and directional control, we visualize different internal quantities calculated by our algorithm—the smoothed short-term powers of the error signal $\bar{e^{2}\left(n\right)}$ (dark blue) and measured sensor signal $\bar{y^{2}\left(n\right)}$ (green), the estimated noise power ${\hat{\sigma }}_{b}^{2}$ (red dotted), and the two different versions of the system distance estimate, $\bar{{\hat{\Gamma }}_{\mathrm{DT}}\left(n\right)}$ (ochre) and $\bar{{\hat{\Gamma }}_{\mathrm{CEA}}\left(n\right)}$ (purple). For reference, we also plot the chosen threshold $\zeta_{\mathrm{suff}}=-78$ dB (grey dash-dotted) used for directional control. In a second axis, we also visualize the time-dependent step size behavior, once more on a logarithmic axis. This can be seen in Figure 9.

Before the initial adaptation start at time 0 s, the noise estimation and settling periods are visible. The estimated noise power ${\hat{\sigma }}_{b}^{2}$ results from the average sensor power y(n) during the first of those. Afterwards, the field around the sensor is already driven at the first position but the adaptation is not yet started. With the start of the adaptation process, a similar behavior to the 1D estimation is visible. At a slightly fluctuating sensor input signal due to the noise excitation, the error and, correspondingly, the system distance estimates drop steeply with the start of adaptation. As before, the CEA estimate decreases below the DT estimate after a while, with the step size following this decreasing trend due to the corresponding step size control. The measurable error levels off more quickly but the system distance can be reduced by the proposed algorithm nonetheless. Most directional steps at the visualized start of the experiment show the directional change to the next position step being triggered by a sufficiently small system distance (according to (34)) with examples for the maximum time threshold (cf. (36)) being visible as well (e.g., the second position). At the start of each new direction, the jump of the step size to 1 for a short period $N_{\mathrm{fix}}$ is clearly visible in the lower axis of Figure 9.

**Figure 10:**
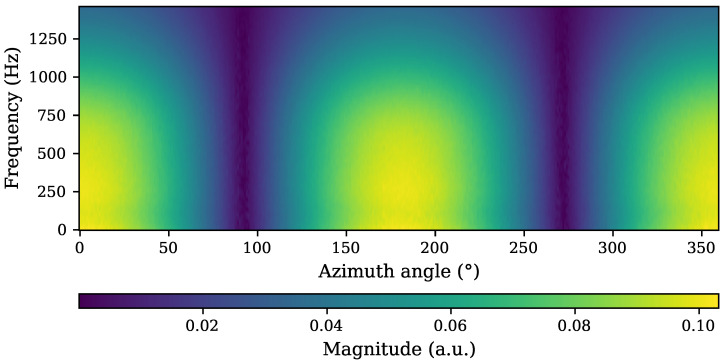
Spectrogram-like visualization of the results of a 2D estimation run. The frequency response is plotted over the 360° measured on the x-axis with the y-axis depicting the frequency bins up to around 1.4 kHz. The color encodes the respective magnitude derived via FFT from the impulse response estimations. The expected behavior with a low-pass characteristic at around 1 kHz and insensitive directions at 90° and 270° is clearly visible.

A full run of our algorithm in the 2D case with the parameters shown in Table 3 took around 461.48 s or 7.69 min. At an angular resolution of 1°, this results in an average of under 1.3 s per direction. The resulting matrix of estimated impulse response coefficients over the angles can be visualized similarly to a spectrogram by calculating the corresponding frequency responses (Figure 10).

The magnitude was calculated from the impulse response estimates by taking the absolute values from a real-valued FFT algorithm (rfft implementation in SciPy [29,30]) with an FFT size of 8192 (zero padding interpolation). As the expected bandwidth of the sensor extends to 1 kHz and the estimate cannot be expected to be accurate above 2 kHz (cf. Section 2.1), we only show the frequency up to around 1.4 kHz on the y-axis. The response is then encoded by a color for the magnitude of a specific bin and is plotted for each of the 360° (x-axis). The low-pass characteristic is clear in and around the sensitive axis (0°, 180°, and 360°), as is the cosine-like behavior with insensitive areas around 90° and 270°.

**Figure 11:**
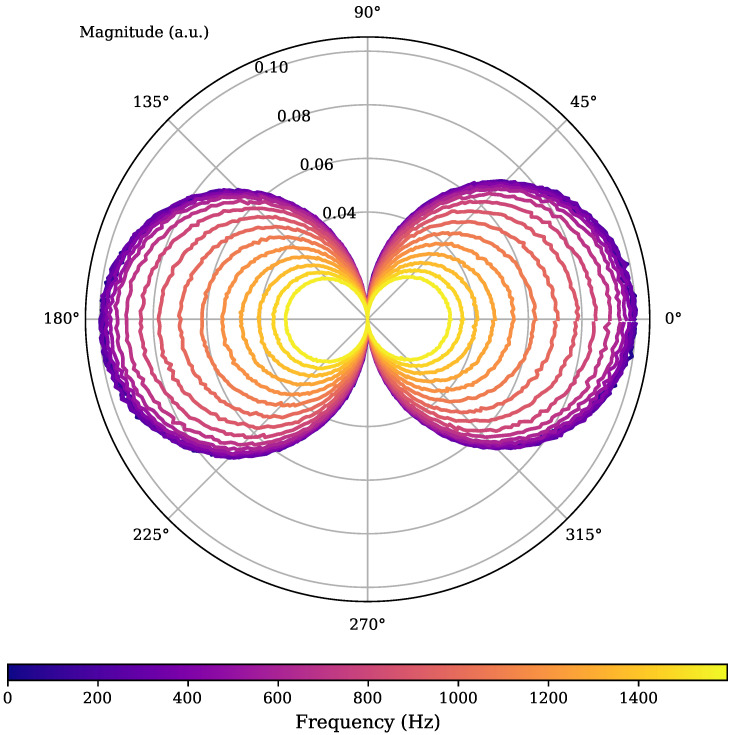
Alternative visualization in a polar plot of the 2D estimation results. The radial component corresponds to the magnitude at the respective angular direction. The color encodes different frequency bins in the estimated frequency response. The expected cosine behavior is clearly apparent and the low-pass characteristic is discernible from the much smaller magnitudes of higher frequency bins.

The directivity behavior is clearly visible when showing singular frequency bins only over the angular directions. This leads to the polar representation plotted in Figure 11. Here, the frequency responses are based on a shorter, 512-sized direct implementation of a magnitude discrete Fourier transform (DFT) that is directly calculated at runtime with the algorithm to provide visual feedback during experiments. Estimated responses from every second bin were chosen and are depicted in the figure, this time being encoded by lighter colors. This shows the directivity pattern in a more tangible fashion, while also providing the low-pass information due to the shrinking radial dimensions at higher frequency bins.

### 3.4. 3D Estimation and Adaptation Times

Lastly, the algorithm was tested for the intended full 3D directivity estimation. We chose a slightly lower resolution of 3° in azimuth and elevation as well as a higher tolerance with regards to the target system distance $\zeta_{\mathrm{suff}}$. We also changed the axis of rotational symmetry to the x-axis, so that the direction of the excitation field looped around the sensor’s sensitive axis.

**Figure 12:**
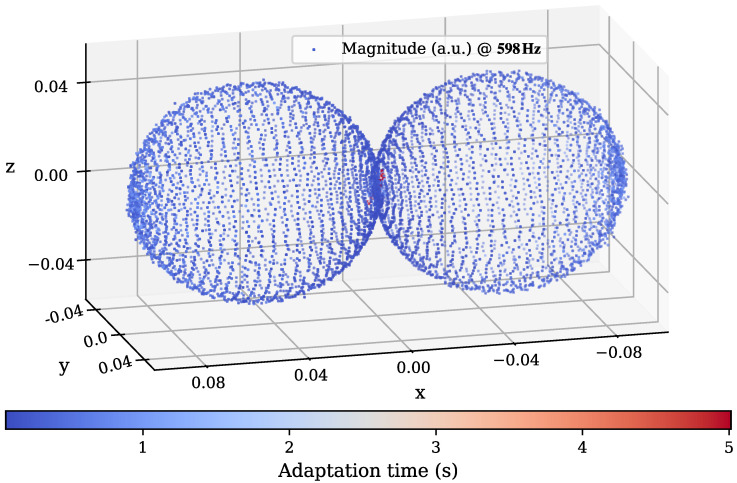
Scatter visualization of the estimation results of a 3D characterization, shown for an exemplary frequency bin in the sensitive range of the sensor at 598 Hz. Each individual point represents a single estimation position, with the radius to the center showing the strength of the magnitude. The color mapping encodes the time taken for adaptation at each field direction before switching to the next direction of interest.

Due to the added dimension, a visualization of only a single frequency bin around 598 Hz is shown in Figure 12. This can be seen as an expansion of the polar plot for the 2D experiment, where the radial distance of a scatter point to the origin corresponds to the estimated magnitude of the sensor at the shown frequency with a field excitation from the radial direction from the origin. In addition, the color in this figure maps the time taken for adaptation to the individual points of estimation. It is visible that only the estimations around the insensitive area show up red with higher adaptation times.

**Figure 13:**
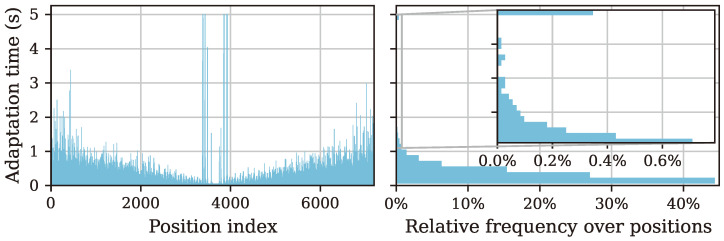
Histogram visualization of the adaptation time over the directivity positions. A stark increase in adaptation times leading up to the sensor’s insensitive direction is apparent, which is mitigated due to the explained fallback thresholds. The relative frequency of different ranges of adaptation times on the right shows an overall cumulation of most positions in the sub-second range.

The overall 3D estimation run took around 2435.1 s or 40.59min. With $N_{\varphi }N_{\theta }=7200$ points, this results in an average of under 0.34 s per direction. This speedup is related to the higher tolerance of the target threshold, but could also be caused partly by the higher overall points with generally faster adaptation. Not only the average adaptation time is of interest but also the times for an individual direction. A visualization of these adaptation times is shown in a position-index-based and histogram form in Figure 13.

Here, we can once more see the increase in adaptation times around the sensor’s insensitive direction because of the low amount of information available to our algorithm due to the bad SNR of the sensor response. When the sensor signal power becomes low enough, the fallback condition (32) is triggered, which leads to minimal adaptation times for these directions (middle). The histogram gathers the individual adaptation times into bins of small ranges, showing a strong dominance of sub-second times. Still, from the spread over the directions, a trend is visible for a higher frequency of slightly longer adaptation times towards the start and the end of the experiment. These correspond to the sensitive areas of the sensor under investigation and were expected to have a higher SNR and thus a better adaptation behavior.

## 4. Discussion

Overall, we were able to show the working principle of the algorithm proposed in this contribution together with the dedicated hardware setup built for it. We achieved the goal of estimating directive sensor system behavior by means of its impulse and frequency response model with relatively high directive resolution at short system runtimes.

A 1D experiment was performed with two different fixed and adaptive step sizes, the latter controlled by DT and CEA, respectively. We found the expected trade-off for fixed step sizes with a larger step size leading to faster convergence and a smaller step size to a smaller remaining system distance. We were also able to achieve fast convergence and smaller resulting system distance estimates with both adaptive control schemes. The better behavior of the CEA control, with the potential to achieve even lower remaining system distance estimates, shows the control approach’s potential and success. However, further investigation of the two distance estimations would be beneficial for a more comprehensive understanding of the algorithm behavior in future work. The DT estimate in this work might also be investigated further with a practically introduced artificial delay instead of relying on assumptions about the sensor ringdown.

The 2D experiment was conducted to check the directional control part of the proposed algorithm. Recorded internal signals show similar behavior for the individual adaptation steps and a working logic to change the excitation to the next planned field direction. With a resolution of 1° and a target system deviation of −78 dB, a full estimation was performed in well under 8 min. We proposed different frequency-, direction-, and sensor response-dependent visualizations for the resulting estimations, which clearly show the algorithms capacity to model the sensor’s cosine-like directivity and low-pass frequency behavior. Due to the chosen design frequency limit for the coil equalizers (2 kHz), the analysis is limited to frequency ranges below this limit.

Lastly, the 3D directivity frequency response estimation targeted in this contribution was realized. With 3° resolution—120 and 60 steps in azimuth and elevation direction, respectively—and a target system distance of −72 dB, the full algorithm run took under 41 min, which is very fast for the amount of directions with full impulse response estimates. Thus, a lot of directional and frequency response information can be gathered with the proposed method. This suggests the exploitability of the correlation of the response of a magnetic sensor to excitation from a directionally similar field. Adaptation times are in general very fast, with a trend of slightly slower adaptation in areas with a larger overall sensor response. A reason for this might be that the sensor’s estimated impulse response $\hat{h}\left(n\right)$ has a higher amplitude for these areas. This might lead to higher remaining noise on the dead-time coefficients and thus a larger DT system distance estimate ${\hat{\Gamma }}_{\mathrm{DT}}\left(n\right)$, preventing the triggering of the next step by the directional control. Spikes in adaptation time close to the sensor’s insensitive direction were expected with an SNR-based threshold in place to mitigate the slowing down of the overall processing. With a different choice of this parameter, the occurrence of these spikes and their respective influence on the overall runtime might be mitigated further.

The presented work naturally has limitations that are not within the scope of this contribution. The conducted experiments were performed only with one sensor, which does not guarantee comparable results with other sensors. The system parametrization (e.g., EQ design) also incorporated a priori knowledge on this investigated sensor type. Further work could investigate the applicability of our findings to magnetic sensors in general. This would include a change of the EQ design to equalize the coil system in the range of the expected sensor bandwidth. Furthermore, other sensor technologies might need adapted parameter sets, e.g., with adjusted filter lengths for a different impulse response behavior, and additional readout electronics to match the devices’ dynamic ranges to the operational ranges of the ADC and DAC components. Otherwise, different converters might also be employed. Generally, the proposed estimation is only expected to cover linear sensor behavior and not possible nonlinear operation points due to the linear nature of the FIR structure adapted by the NLMS algorithm. To estimate nonlinear behavior, a different adaptation structure of, e.g., a Volterra series would be necessary.

The proposed equalization is limited in accordance with the generating coils and cross talk between coils cannot be ruled out entirely. Thus, there might be a deviation between the calculated, ideal excitation field direction and the field the sensor is practically exposed to. As the setup actively generates a field, it is not in a shielded environment and is thus limited to uses with magnetic sensors working in unshielded conditions. This relates to the additional impact of the potential influence of external fields, e.g., Earth’s magnetic field or stray fields by nearby equipment, on the sensor’s output. We do not assume a large impact due to the significantly higher strength of the applied fields. Further research is needed to assess possibly introduced low-frequency drifts and similar possible implications. However, this is beyond the scope of this proof-of-principle study.

At the current stage, the resulting impulse and derived frequency response estimates give a qualitative insight into the sensor behavior. They do not contain explicit information about the sensor sensitivity as the goal for this contribution is focused on a qualitative (not quantitative) description.

To convert this into absolute sensitivity measurements, a reference sensor with known sensitivity could be used with identical field actuation in one direction. From such knowledge of the absolute field, the arbitrary units could be related to quantitative sensitivity measures. A different approach could be the deviation of theoretical field strengths based on the Helmholtz coil equations (cf. (2)) with measured currents through the coils.

The runtime of the multidimensional estimations of the algorithm and the estimated quality (given by the system distance) of the respective results depend on the chosen parameters. More investigated field directions and a smaller target system distance can lead to longer runtimes, with a possible gain of higher-quality results. A trade-off must be made depending on the individual objectives and overall conditions of a measurement. A quantitative analysis of the influence of different algorithm parameters could be studied with a potential investigation of optimization procedures for ideal parameter combinations for distinct objectives.

## 5. Conclusions and Outlook

In this paper, we introduced a novel magnetic sensor characterization scheme based on adaptive filters. Our algorithmic approach uses an advanced CEA step size control to achieve fast and precise convergence of the NLMS algorithm for an impulse response estimation. We also implemented several threshold-based control mechanisms to steer a magnetic field generated by a self-built 3D Helmholtz coil setup for an automatic estimation procedure of 3D directive impulse response behavior. The resulting full system was used for 1D, 2D, and 3D estimation experiments. Due to the scope of this proof-of-concept contribution, some limitations remain, e.g., the qualitative nature of the resulting sensor response estimates without explicit knowledge of the sensitivities.

The system presented in this contribution provides a foundation for the future possibility of time-efficient, directivity-dependent analysis of magnetic sensors and, therefore, high-throughput characterization of changing devices in research or production environments, for example, in medicine. This could significantly impact application research dealing with sensors lacking the desired characterization information, especially where this is needed for solving, e.g., inverse problems that could use the results for respective modeling [31]. Overall, this could lead to more sophisticated models in applications depending on the directivity information of magnetic sensors and, consequently, to increased accuracy and development speed.

## Figures and Tables

**Table 1:** Coil parameters.

Coil	Resistance (DC)	Edge Length *ℓ*
Inner—x	2.5 Ω	306 mm
Mid—y	3.0 Ω	352 mm
Outer—z	3.5 Ω	398 mm

**Table 2:** Equalizer parameters.

Equalization Target Coil	Normalization Impedance $Z_{0}$	Resulting Gain Factor β
Inner—x	62.1076 Ω	1.0
Mid—y	62.3548 Ω	1.1549
Outer—z	54.5292 Ω	1.1419

**Table 3:** Collection of algorithm parameters in the experiments. Values in colored cells are used in all indicated experiments.

Parameter	Symbol	Value		
		1D	2D	3D
Frameshift	$N_{\mathrm{frame}}$	512 (10.67 ms)		
Filter length	$N_{\mathrm{filter}}$	20 ms		
Noise estimation period	$N_{b}$	1 s		
Initial settling period	$N_{\mathrm{set}}$	1 s		
Time period with fix step size	$N_{\mathrm{fix}}$	32 ms		
Smoothing factor rising	$\gamma_{r}$	0.997		
Smoothing factor falling	$\gamma_{f}$	0.999		
DT estimation interval	$N_{\mathrm{DT}}$	200 (4.17 ms)		
Adaptive control threshold	$\eta_{\mathrm{fix}}$	−10 dB		
CEA control threshold	$\eta_{\mathrm{DT}}$	−15 dB		
Minimum time threshold	$N_{\min}$	30 s	50 ms	
Maximum time threshold	$N_{\max}$	30 s	5 s	
Noise offset threshold	$\zeta_{\mathrm{SNR}}$	—	27 dB	
Number of elevation steps	$N_{\theta }$	1		60
Number of azimuth steps	$N_{\varphi }$	1	360	120
System distance quality threshold	$\zeta_{\mathrm{suff}}$	—	−78 dB	−72 dB

## Data Availability

The raw data supporting the conclusions of this article will be made available by the authors on request.

## Abbreviations

The following abbreviations are used in this manuscript:

1D	One-dimensional
2D	Two-dimensional
3D	Three-dimensional
ADC	Analog–digital converter
CEA	Contraction–expansion approach
CPSD	Cross power spectral density
CRC	Collaborative Research Centre
DAC	Digital–analog converter
DC	Direct current
DFT	Discrete Fourier transform
DT	Dead time
EQ	Equalizer
FFT	Fast Fourier transform
FIR	Finite impulse response
IIR	Infinite impulse response
ME	Magnetoelectric
MR	Magnetoresistive
NLMS	Normalized least mean squares
OPM	Optically pumped magnetometer
PSD	Power spectral density
SNR	Signal-to-noise ratio
SQUID	Superconducting quantum interference device
